# QSI and DTI of Inherited White Matter Disorders in Rat Spinal Cord: Early Detection and Comparison with Quantitative Electron Microscopy Findings

**DOI:** 10.3390/diagnostics15070837

**Published:** 2025-03-25

**Authors:** Maysa Teixeira Resende, Benjamin K. August, Daniel Z. Radecki, Madelyn Reilly, Abigail Komro, John Svaren, Debbie Anaby, Ian D. Duncan, Yoram Cohen

**Affiliations:** 1 School of Chemistry, The Sackler Faculty of Exact Sciences, Tel Aviv University, Tel Aviv 699781, Israel; maysateixeira.enq@gmail.com; 2 School of Veterinary Medicine, University of Wisconsin-Madison, Madison, WI 53706, USAian.duncan1314@gmail.com (I.D.D.); 3 Waisman Center, University of Wisconsin-Madison, Madison, WI 53706, USA; 4 Department of Diagnostic Imaging, Sheba Medical Center, Ramat Gan 52620, Israel; 5 The Sackler Faculty of Medicine, Tel Aviv University, Tel Aviv 699781, Israel; 6 The Sagol School of Neuroscience, Tel Aviv University, Tel Aviv 699781, Israel

**Keywords:** diffusion MRI, q-space diffusion MRI, QSI, spinal cord, white matter, axon diameter, myelin disorders, rat mutants

## Abstract

**Background:** Inherited white matter (WM) disorders of the central nervous systems (CNS), or leukodystrophies, are devastating diseases that primarily affect children, many of whom die early in life or suffer from long-term disability. **Methods**: q-Space diffusion MR imaging (QSI) and diffusion tensor MR imaging (DTI) with the same resolution and timing parameters were used to study the spinal cords (SCs) of two myelin mutants that are experimental models of WM diseases of different severity, namely the 28-day-old *taiep* and Long–Evans Shaker *(les*) rats. The aim was to verify if and which of the diffusion methodologies used is more suitable for early detection of the milder *taiep* pathology and to characterize its early phase. We also aimed to compare the diffusion MRI results with quantitative electron microscopy (EM) findings. **Results**: We found that at this early age (28 days), both QSI and DTI were able to detect the severe *les* WM pathology, while the milder WM pathology in the SC of the *taiep* rats was detected only by QSI. An increase in the mean radial displacement (RaDis), representing the MRI axon diameter (AD), and a decrease in the probability for zero displacement (PZD) were observed in the dorsal column (ROI 1) of the *taiep* SCs. In other WM areas, the same trends were observed but the differences were not of statistical significance. In DTI, we found some lower fractional anisotropy (FA) values in the *taiep* SCs compared to the controls; however, these differences were not statistically significant. For the more severe *les* pathology, we observed a dramatic increase in the RaDis values and a large decrease in PZD values in all ROIs examined. There, even the FA values were lower than that of the control SCs in all ROIs, albeit with much smaller statistical significance. These MRI results, which show a higher detectability of WM pathology with heavier diffusion weighting, followed histological findings that showed significant myelin deficiency in the dorsal column in the *taiep* SCs and a practically complete myelin loss in all WM areas in the *les* SCs. This study also revealed that, under the experimental conditions used here, the apparent increase in RaDis agrees better with myelin thickness and not with average AD extracted form EM, probably reflecting the effect of water exchange. **Conclusions:** These results, corroborated by diffusion time-dependent QSI, also imply that while diffusion MRI in general and QSI in particular provide acceptable apparent axon diameter estimations in heathy and mature WM, this appears not to be the case in severely damaged WM where exchange appears to play a more important role.

## 1. Introduction

Leukodystrophies are devastating genetic disorders of white matter (WM) of the central nervous system (CNS) that affect the quality of life of many children and adults, and can result in long-term disability or death [1,2,3,4]. These WM disorders are related to abnormal myelin production (hypomyelination or dysmyelination) or myelin loss (demyelination). In addition to these inherited disorders, acquired WM disorders may result from trauma, immune-mediated, viral, metabolic or toxic causes [4]. Indeed, many severe neurological diseases in humans result from disorders of WM and a lack or loss of myelin, ranging from the most common WM disease, multiple sclerosis (MS), to the rarer genetic leukodystrophies [3,4,5]. Leukodystrophies constitute a large, highly heterogeneous group of genetic diseases which have become more frequently identified and characterized in recent years as a result of advances in gene sequencing [2,3] and the improvement in MRI detectability [6]. Despite these advances in the diagnosis of leukodystrophies, therapy is still lagging [2,3]. As amelioration in the disease course might only be achievable with early therapeutic intervention, there is an urgent need for more efficient, early and unequivocal diagnosis of these disorders [2].

Inherited disorders of myelin also occur spontaneously in animals [4]. Some of these myelin mutants have similar mutations and myelin pathologies associated with human leukodystrophy disorders, and therefore can be used as animal models of these devastating neurological diseases. The list of WM mutants encompasses mutations in both proteolipid protein (*PLP1*) and myelin basic protein (*MBP*) genes and other myelin-associated genes [4,5]. The myelin-deficient (*md*) rat has an X-linked mutation in the *PLP1* gene and exhibits a very severe and lethal disease, surviving for only 24 to 28 days. [4,5]. The Long–Evans Shaker (*les*) rat is an autosomal recessive mutant that has a mutation in the MBP-encoding gene [7]. The *les* rat exhibits both dysmyelination and demyelination processes but can survive, with husbandry support, for 9 to 12 months in contrast to the *md* rat [6,7,8]. The *les* rat, has extensive, though rudimentary myelination of many spinal cord axons at two weeks of age, but by 4–8 weeks of age practically all this myelin is lost [5,8]. The *taiep* rat was discovered in 1989 as a spontaneous mutation in a colony of Sprague Dawley rats [9]. The *taiep* rat is also an autosomal recessive mutant, characterized by initial hypomyelination of the CNS followed by demyelination of certain spinal cord tracts and much of the brain. The *taiep* rat develops a locomotor syndrome characterized by tremor (t), ataxia (a), immobility (i), epilepsy (e) and paralysis (p) [10,11], which result from a mutation in the *TUBB4a* gene [12]. The *taiep* rat is a model of the rare human leukodystrophy known as Hypomyelination with Atrophy of the Basal Ganglia and Cerebellum (HABC) [12,13]. The uniqueness of the *taiep* rat is that affected rats can survive, with little assistance, for up to two years of age, but with gradually progressive neurologic dysfunction. Despite extensive characterization of the neurobiology of the *taiep* rat (4), the WM abnormalities have never been studied by diffusion magnetic resonance imaging (MRI).

MRI is perhaps the most important imaging modality for soft tissues and diffusion MRI methodologies are paramount for imaging of the CNS and have been widely used to study diseased and healthy WM [14,15]. The unequivocal demonstration that water diffusion in WM is highly anisotropic [16] and the subsequent development of diffusion tensor imaging (DTI) led to the clinical advancement of MRI [14,15,17]. Despite some debate [18], it appears that water diffusion anisotropy in WM originates from tissue’s microstructural elements such as axonal membranes, myelin sheaths, microtubules and more [19], although the relative contribution of each is difficult to determine and may even vary in different pathologies [19,20,21]. More than 25 years ago, it was demonstrated that at sufficiently high diffusion weighting (high b values), water signal decay in neuronal tissues is not mono-exponential [22,23,24] implying that proper analysis of such data should, in principle, provide more detailed and specific structural information on the investigated tissues. Model-free approaches such as high b-value q-space diffusion imaging (QSI) [25,26,27,28,29] and then diffusion kurtosis imaging (DKI) [30,31,32] were developed for obtaining microstructural information from such MRI data. This was followed by the development of numerous model-based approaches to obtain the same goal [33,34,35,36,37]. In the last decade, more efforts have been directed to expanding the acquisition schemes and modelling from single to double diffusion encoding MR experiments [38,39,40,41] and evaluating the benefit of using oscillating gradients [42,43].

DTI has been used to study the spinal cords of different mutants [44,45,46,47,48,49,50,51]. We have used QSI to study the CNS of the *md* rat [52,53,54], and more recently, the CNS of the *les* rat [55]. Based on these and other studies [44,45,46,47,48,49,50,51,52,53,54,55,56,57,58,59], it was concluded that, generally, an increase in the radial and mean diffusivities (RaD and MD, respectively) concomitantly with a decrease in FA values is indicative of myelin damage. A decrease in MD and axial diffusivity (AxD) concomitant with a decrease in FA values, however, appears to be more related to axonal damage [19,58,59]. It is important to note, however, that the relative contribution of each of these structural components of WM to the computed MRI indices remain difficult to determine and may change and differ in each pathology and even with the severity of a specific disease. However, in all our previous studies of WM mutants, MRI was compared to gross histology focusing on the existence and absence of myelin [53,55].

In the present study, we used QSI and DTI, collected with the same resolution and timing parameters, to study, ex vivo, the spinal cords of 28-day-old *taiep* and *les* mutants and their normal littermates. The microstructural information obtained from MRI experiments were compared, for the first time, with detailed quantitative electron microscopy (EM) data. The aims were to test which of the diffusion MRI methods could detect the much milder *taiep* pathology at this early stage. In addition, we planned to image the *taiep* SC pathology for the first time by diffusion MRI and to compare the *taiep* and *les* microstructural information obtained from diffusion MRI with detailed EM structural findings collected on the SCs of the same ROI of the same mutants. This study not only tests the ability of QSI and DTI to discriminate between myelin mutants that differ in the severity of their WM pathology at an early stage of the disease, but also provides a comparison between the microstructural information obtained from QSI and quantitative EM of normal and diseased SCs. The present study provides insights into the microstructural features associated with changes in the extracted diffusion MRI indices.

## 2. Materials and Methods

### 2.1. Sample Preparation

The rodent study protocol (V005423) was reviewed and was approved on 28 February 2019 by the School of Veterinary Medicine Animal Care and Use Committee of the University of Wisconsin–Madison. Twenty animals, including five *taiep* and five *les* rats and their normal littermates (five in each group, i.e., *n* = 5), were used in this study. The 28-day-old rats were perfused with 0.1 mM phosphate-buffered saline, followed by 2.5% glutaraldehyde buffered in 0.1 mM phosphate-buffered saline. Following perfusion, spinal cords were excised and stored in 2.5% glutaraldehyde and then shipped to Tel Aviv. The fixed tissues were stored at 4 °C. Twenty-four hours before the MRI experiments, the samples were immersed in phosphate-buffered saline solution to remove the glutaraldehyde. The samples were gently dried and then mounted in 4 mm sleeves which were then inserted into 5 mm NMR tubes filled with Fluorinert (Sigma, Saint Louis, MI, USA), with their long axis parallel to the z direction (the B_0_ direction) of the magnet. The temperature was maintained at 25 ± 0.2 °C throughout the MRI experiments.

### 2.2. Ex Vivo MRI Experiments

MRI experiments on *les*, *taiep* and their respective control spinal cords were performed using a 9.4T spectrometer (Bruker, Karlsruhe, Germany) equipped with a micro5 imaging probe and gradient systems (Bruker, Karlsruhe, Germany) capable of producing pulsed field gradients of up to 3000 mTm^–1^. The DTI and QSI experiments were performed using a single-shot spin-echo EPI imaging sequence. The field of view was 6.4 × 6.4 mm collected with a 64 × 64 matrix, no zero filling applied, resulting in an in-plane resolution of 100 × 100 microns. Three contiguous slices in the cervical cord with a slice thickness of 2 mm were collected with the following timing parameters: TR = 2000 ms, TE = 65 ms, δ = 2 ms and ∆ = 50 ms. In the QSI experiments, the diffusion gradients were incremented up to 1600 mT/m in 16 equal steps, resulting in maximal *b* and *q* values of 31,967 s/mm^2^ and 1362 cm^−1^, respectively. In all QSI experiments, the diffusion gradients were perpendicular to the long axis of the spinal cord. The number of averages (NA) was 50, resulting in a collection time of 0.5 h. An in-house MATLAB program (version R2021b) was used for image analysis of the QSI data as described previously by us [26,28]. This analysis provided the radial displacement (RaDis) map representing the MRI average axon diameter (AAD) and the probability for zero displacement (PZD) map [26,28,60]. Note that the QSI RaDis and AAD were computed as 0.425 of the width at half-height of the displacement distribution profile as suggested by Cory and Garroway [60]. DTI experiments were carried out with the above parameter in 19 directions and with *b* values of 0, 500, 1000, 1500 and 2000 s/mm^2^. We analyzed the data up to 1500 s/mm^2^ after observing that analyzing the entire data set in fact increased the standard error of the mean (SEM) of the extracted FA values. Again, the NA was 50, resulting in a total acquisition time of 2.0 h. This was undertaken to ensure high and similar signal-to-noise ratios (SNRs) for both methods, at least at *b*_0._ ExploreDTI [61] (version 4.8.6) was used to analyze the DTI data.

To evaluate the effect of exchange, a series of QSI experiments were performed on 5 different SCs with exactly the same protocol and experimental parameters, the only differences being that the TE was set to 90 ms and the diffusion time to 30, 50 or 75 ms.

### 2.3. MRI ROIs Analysis and Statistics

The quantitative analysis of the data was performed on three selected regions of interest (ROIs) in the rats’ spinal cords. We decided to use specific ROI analysis rather than global voxel-based analysis, to allow, as much as possible, spatial correspondence between MRI and EM microstructural information. As we could not perform quantitative EM on the entire SCs of the different mutants and their littermates, we selected three ROIs (from the dorsal, lateral and ventral columns) where some changes were expected based on previous histological studies of *taiep* SCs [4,5,13]. For each ROI we extracted the average RaDis and PZD from QSI. From the DTI data, we computed the average FA for each ROI. Student’s *t*-test was used for statistical analysis and *p*-values equal to or less than 0.05 was considered statistically significant.

### 2.4. Samples for Light and Electron Microscopy

The rat spinal cords were post-fixed overnight in 2.5% buffered glutaraldehyde following intravascular perfusion. A 2 mm block that corresponded to the adjacent piece of the cord, sent to and imaged in Tel Aviv, was trimmed and processed for light and electron microscopy. The tissues were post-fixed in 1% osmium tetroxide and dehydrated with a series of ethanol solutions. Propylene oxide was used as a transitional fluid for resin infiltration and embedding. We first prepared 1-micron sections that were stained with toluidine blue, then ultrathin sections were cut and mounted on copper grids (Electron Microscopy Sciences, Hatfield, PA, USA) and stained with uranyl acetate, followed by lead citrate. Images were taken from the dorsal (RO1), lateral (RO2) and ventral (RO3) columns. Images were captured on a Philips EM 120 with AMT Biosprint digital camera (Philips, Amsterdam, The Netherlands) housed at the EM Laboratory of the School of Public Health and Medicine, University of Wisconsin–Madison. Within each specific area of interest, grid squares (11,000 um^2^/square) were used as a visual tool to select individual fields of 1–3 images (per grid square) to ensure no field selection overlaps occurred.

Quantification that included myelin thickness and axon diameter measurements was completed using ImageJ version 1.530 (available in the public domain at http://imagej.nih.gov/ij/ accessed on 16 February 2025 by the NIH, Bethesda, MD, USA) software, where each image magnification was calibrated, and a thresholding was utilized to automatically select areas inside the electron-dense myelin. Measurements of 150–250 axons were used for the results reported for each individual area sampled. The mean (±SEM) of these values were computed for each ROI and compared with the mean values extracted from MRI data

## 3. Results

### 3.1. Diffusion MRI Findings in the taiep and les SCs

**Figure 1:**
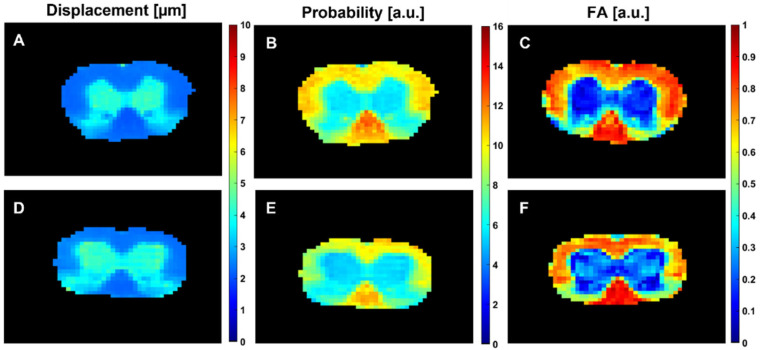
Radial displacement (RaDis) (**A**,**D**), PZD (**B**,**E**) and FA (**C**,**F**) maps of the spinal cords of 28-day-old control (**A**–**C**) and *taiep* (**D**–**F**) rats. The radial displacement and PZD maps were obtained from QSI experiments, collected with the following parameters: *q_max_* = 1362 cm^−1^ (*b_max_* = 31,967 s/mm^2^), δ = 2 ms, ∆ = 50 ms, TE = 65 ms, NA = 50 and a total acquisition time of 0.5 h. The FA values were obtained from spin-echo DTI experiments, collected with the following parameters: 19 directions, *b* values of 0, 500, 1000 and 1500 s/mm^2^, δ = 2 ms, ∆ = 50 ms, TE = 65 ms, NA = 50 and a total acquisition time of ~2 h.

Figure 1A,D,B,E show the radial displacement (RaDis) and the probability for zero displacement (PZD) maps, extracted from QSI experiments for the spinal cords of representative 28-day-old control (Figure 1A,B) and *taiep* (Figure 1D,E) rats. Note that the QSI experiments were performed perpendicular to the long axis of SC, making the average RaDis measured a good estimate of the average AD (AAD). Figure 1C,F present the FA maps, extracted from DTI experiments performed on the same SCs. We decided to use the FA, which is the more commonly used DTI parameter, and because it is also the most robust DTI parameter when studying ex vivo systems that are stored in aqueous formalin solutions and need to be washed by saline prior to each MRI inspection.

The MRI maps presented in Figure 1 show very pronounced WM and grey matter (GM) contrast. In WM, lower RaDis (~2–3 μm) and higher PZD values of about 10–12 a.u., as well as higher FA of about 0.8 (a.u.) compared to GM areas were seen, as expected. In GM the RaDis, PZD and FA were about 4–5 μm, 6–7 (a.u.) and 0.1–0.2 (a.u.), respectively. These observations are to be expected since water diffusion is more restricted perpendicular to the long axis of the axons in the WM of the SC, making water diffusion more anisotropic in WM compared to GM [14,15,16,19,21]. A closer inspection of the QSI indices of the WM of the control and the *taiep* SCs, presented in Figure 1, indicates that, at least in the dorsal column of the *taiep* rat SC, the RaDis values are higher than that of the control SCs, while the PZD values of this region are lower. In contrast, it is difficult to observe any differences between the QSI indices of other WM regions without quantitative analysis. For the FA maps obtained from DTI, presented in Figure 1C,F, it is difficult to detect any changes in the different areas of the WM of the same SCs.

**Figure 2:**
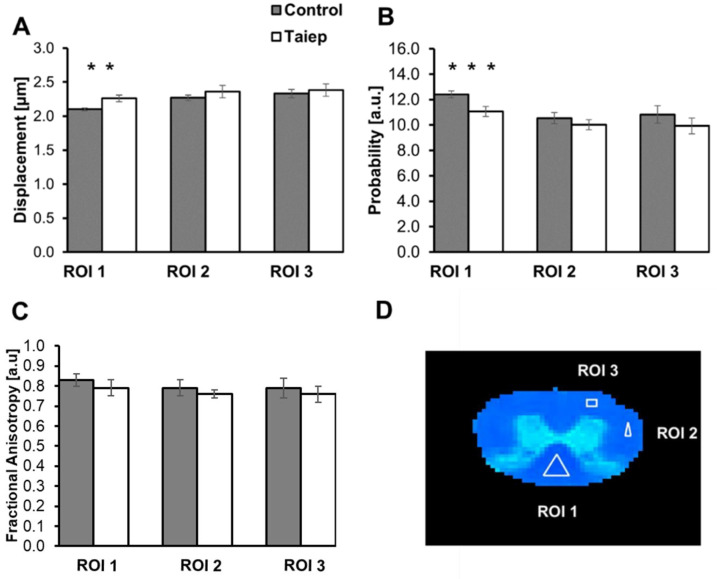
Average radial displacement (RaDis) (**A**), PZD (**B**) and FA (**C**) values for the SCs of 28-day-old control (grey columns) and *taiep* rats (white columns) in the three ROIs defined in (**D**). Five samples were analyzed in each group (*n* = 5). ** *p* < 0.01, *** *p* < 0.001.

For a quantitative evaluation of MRI indices and for more direct comparison of MRI results with EM, we chose to analyze three ROIs: one in the dorsal column (ROI 1), one in the lateral column (ROI 2) and another in the ventral column (ROI 3). The results of such an analysis are presented in Figure 2 and in Table A1. Table A1 shows the QSI indices and the FA values for each SC in the two groups, their group means (±SEM) and the statistical significance of the results between the two groups. Figure 2 presents, graphically, the comparison between the average MRI indices for the three ROIs between the *taiep* and control SCs along the definition of the three ROIs analyzed (Figure 2D).

Figure 2 shows that there is an increase in the extracted RaDis and a decrease in the PZD values in the SCs of *taiep* rats as compared to controls, but these changes are, as expected, statistically significant only for ROI 1, i.e., in the dorsal column. In previous histological studies of the *taiep* SCs, the dorsal column was found to be the most affected area in the *taiep* SCs at this stage of the disease [4,5]. For ROI 2 and ROI 3, we observe the same trends but there the changes are not statistically significant. As for the FA, we see a decreasing trend in the *taiep* SCs compared to the control SCs (Figure 2C), but these differences are not statistically significant for all the ROIs analyzed (see also Table A1).

**Figure 3:**
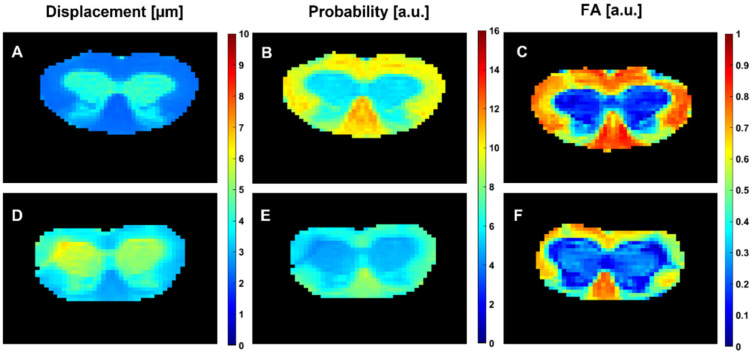
Radial displacement (RaDis) (**A**,**D**), PZD (**B**,**E**) and FA (**C**,**F**) maps of the spinal cords of 28-day-old control (**A**–**C**) and *les* (**D**–**F**) rats. The displacement and PZD maps were obtained from QSI experiments, collected with the following parameters: *q_max_* = 1362 cm^−1^ (*b_max_* = 31,967 s/mm^2^), δ = 2 ms, ∆ = 50 ms, TE = 65 ms, NA = 50 and a total acquisition time of 0.5 h. The FA values were obtained from spin-echo DTI experiments, collected with the following parameters: 19 directions, *b* values of 0, 500, 1000 and 1500 s/mm^2^, δ = 2 ms, ∆ = 50 ms, TE = 65 ms, NA = 50 and a total acquisition time of ~2 h.

Figure 3 shows the same data as presented in Figure 1, but for representative 28-day-old control and *les* SCs. The *les* rats have a much more severe WM pathology than the *taiep* rats at this age [4,5] and this fact is clearly apparent in the MRI maps presented in this figure. Indeed, the WM differences between the *les* and the control SCs are clearly apparent in the QSI maps presented in Figure 3 and can also be seen in some of the WM areas in the DTI maps presented in Figure 3C,F. Clearly, the differences between the controls and the *les* SCs are larger than between the *taiep* SCs and their littermates.

**Figure 4:**
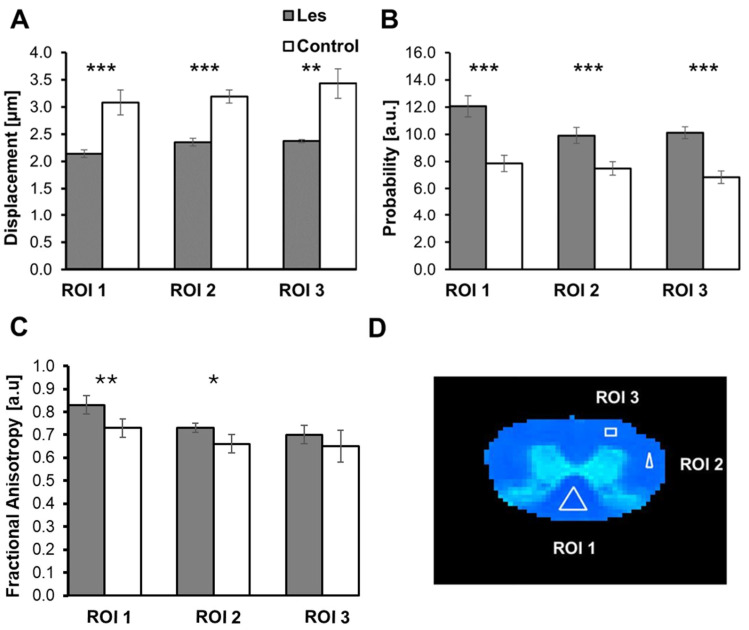
Average radial displacement (RaDis) (**A**), PZD (**B**) and FA (**C**) values for the SCs of 28-day-old control (grey columns) and *les* rats (white columns) in the three ROIs defined in (**D**). Five samples were analyzed in each group (*n* = 5). * *p* < 0.05, ** *p* < 0.01, *** *p* < 0.001.

Figure 4 and Table A2 show the same data as in Figure 2 and Table A1 for the same ROIs but for the SCs of *les* rats and their controls. These data show, unequivocally, that the differences in the QSI and DTI indices in the *les* pathology show the same trends as in the *taiep* SCs, but these differences are much more pronounced in the case of the *les* pathology as compared to the *taiep* pathology. In fact, an increase of about 50% is observed in the RaDis in the *les* SCs in all ROIs analyzed, making these differences highly statistically significant. The average FA values of the *les* SCs are about 10% lower than that of their age-matched controls and were found to be statistically significant in ROI 1 and ROI 2.

### 3.2. EM Findings in the Dorsal, Lateral and Ventral Columns of the taiep and les SC

**Figure 5:**
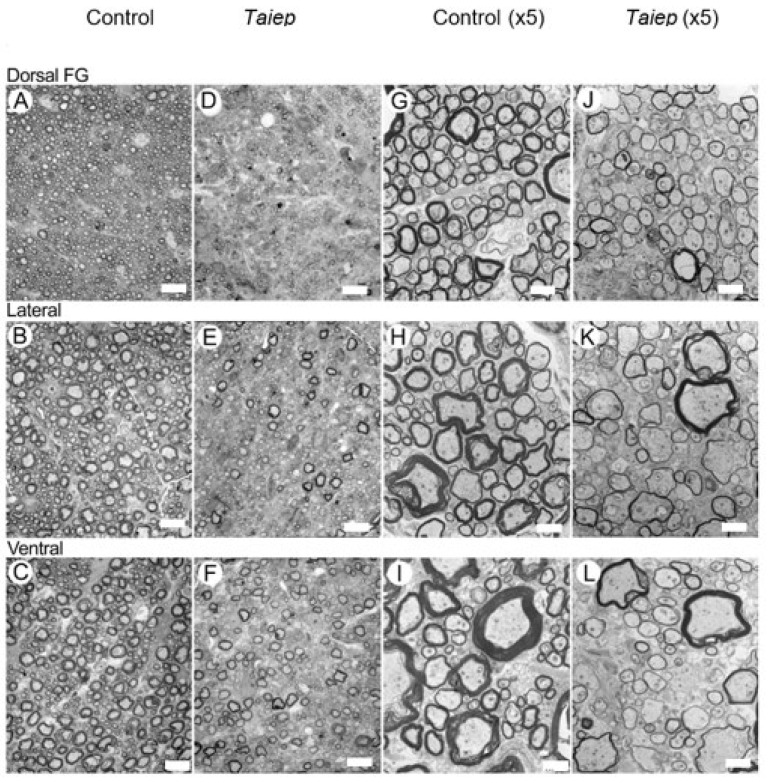
EM montage from the cervical spinal cord adjacent to the section of cord studied by MRI, from 28-day-old normal (**A**–**C**,**G**–**I**) and *taiep* (**D**–**F**,**J**–**L**) rats. The areas illustrated are from the *fasciculus gracilis* of the dorsal column (**A**,**D**,**G**,**J**), lateral column (**B**,**E**,**H**,**K**) and ventral column (**C**,**F**,**I**,**L**). The MRI ROI 1 is in the dorsal column, ROI 2 is in the lateral column and ROI 3 is in the ventral column. The scale bars at (**A**–**F**) and (**G**–**L**) are of 10 and 2 microns, respectively.

**Figure 6:**
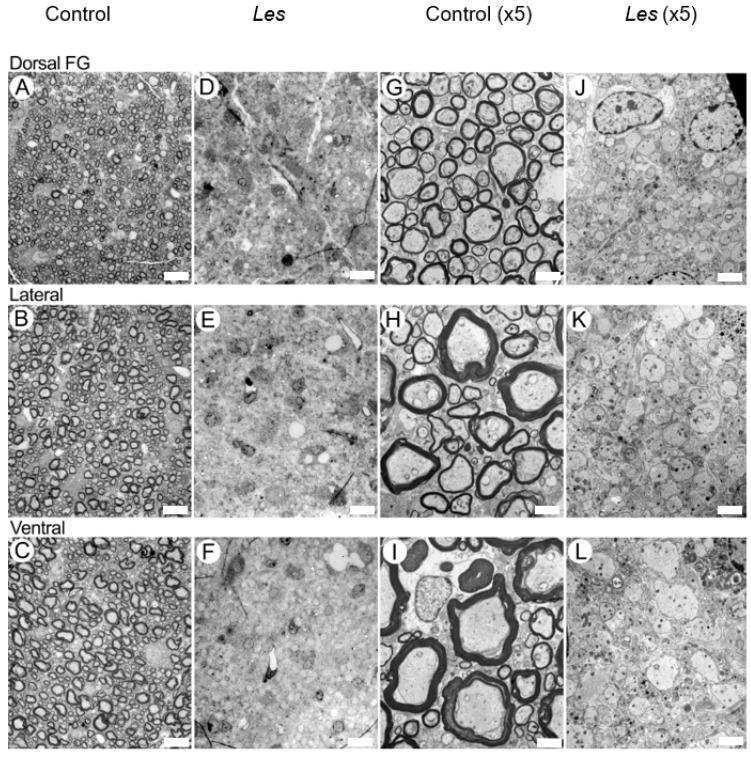
EM montage from the cervical spinal cord adjacent to the section of the cord studied by MRI, from 28-day old normal (**A**–**C**,**G**–**I**) and *les* (**D**–**F**,**J**–**L**) rats. The areas illustrated are from the *fasciculus gracilis* of the dorsal column (**A**,**D**,**G**,**J**), lateral column (**B**,**E**,**H**,**K**) and ventral column (**C**,**F**,**I**,**L**). The MRI ROI 1 is in the dorsal column, ROI 2 is in the lateral column and ROI 3 is in the ventral column. The scale bars at (**A**–**F**) and (**G**–**L**) are of 10 and 2 microns, respectively.

Figure 5 presents EM images from representative SCs of a 28-day-old *taiep* (Figure 5D–F,J–L) and of a control (Figure 5A–C,G–I) rat at two magnifications for the three ROIs studied by MRI. The data shown are for ROIs in the FG in the dorsal column (ROI 1), the lateral column (ROI 2) and the ventral column (ROI 3). Figure 6 shows similar data but for the SCs of a 28-day-old *les* rat and its littermate.

**Figure 7:**
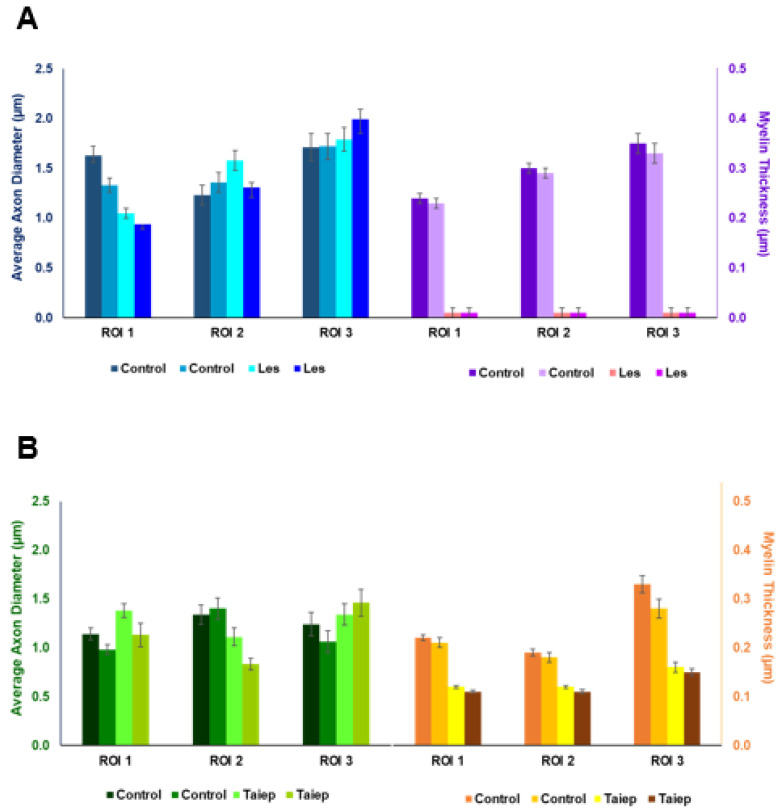
The average axon diameter (AAD) and myelin thickness (in μm) of two control and two diseased SCs as obtained from EM for the three ROIs analyzed by MRI, (**A**) for the *les* SCs and their controls (upper panel), and (**B**) for the *taiep* SCs and their controls (lower panel). Note that in (**A**), the myelin thickness could not be determined for the *les* SCs and was set to zero.

Inspection of the EM data presented in Figure 5 shows that most of the axons in the *taiep* SCs are myelinated, although with a thinner myelin sheath, implying that the g-ratio is higher in *taiep* SCs compared to those of their littermates. From this histological raw data, however, it is difficult to determine if and in which ROI there is a change in the average axon diameter (AAD). A quantitative analysis of the EM findings, presented in Figure 7B and Table A3, shows that in the *taiep* SCs there is some decrease in the AADs in ROIs 1 and 2 in the dorsal and lateral columns and a small increase in the AADs in ROI 3 in the ventral columns as compared to their controls. However, these differences are not statistically significant. These histological results differ from the results observed by QSI regarding AADs. Interestingly, in EM, we observe a significant decrease in the myelin thickness in the *taiep* SCs as compared to the controls in all ROIs investigated (Figure 7B). The decrease in the myelin thickness amounts to 45.5%, 36.8% and 48.4% in the dorsal (ROI 1), lateral (ROI 2) and ventral (ROI 3) columns, respectively.

Figure 6 shows, in contrast, that the *les* SC axons are not myelinated, which results in a g-ratio of about 1.0. Indeed, in these areas with a complete loss of myelin, the changes in the QSI indices are large, and even the changes in the FA extracted from DTI are statistically significant, at least in ROI 1 and ROI 2. Interestingly, it is difficult to determine if there is also an increase in the average axon diameter.

Quantitative analysis of the EM findings, presented in Figure 7A and Table A3, of the three ROIs analyzed by MRI shows that indeed, there are only relatively small, and statistically insignificant, changes in the average axon diameter in ROI 2 and ROI 3 of the SCs of the *les* group compared to their age-matched controls. From EM histology, we found a decrease in the average axon diameter for ROI 1 and nearly no change for ROI 2 in the lateral column (Figure 7A). EM histology, however, shows some small increase in the AAD in ROI 3 in the ventral column, suggesting a lack of agreement between EM and the apparent MRI indices that showed significant increases in the AADs of all ROI analyzed in the *les* SCs.

**Figure 8:**
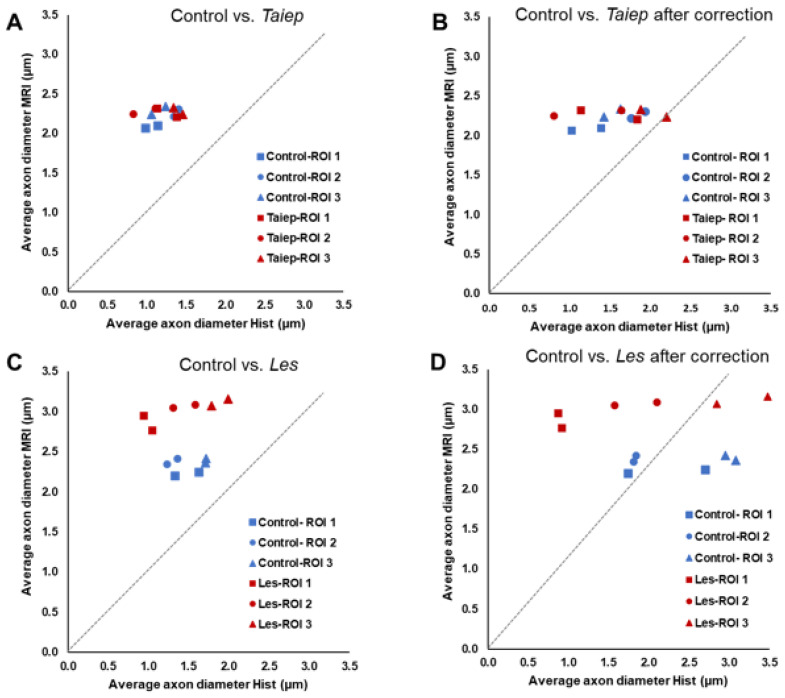
The average axon diameter (AAD) as obtained from QSI and EM histology for the *taiep* SCs and their controls (**A**,**B**), and the *les* SCs and their controls (**C**,**D**), both before (**A**,**C**) and after correction for the number of spins of the EM data, i.e., (cAAD) (**B**,**D**). The cAAD values were computed by dividing the axon diameter from EM by 2 to obtain the axon radius *r*. Then, assuming cylindrical geometry of the axons, we computed the *πr*^2^ values for each axon. The values were summed for all axons in the ROI and divided by *n*, the number of axons, and the result was multiplied by 2 to give the cAAD of the EM data.

Figure 8 shows the comparison between the AADs extracted from the QSI data and from EM both before (Figure 8A,C) and after (Figure 8B,D) the correction of EM diameter values into number of spins for the three ROIs of all four groups of SCs studied. The correction of the AAD values extracted from EM into actual number of spins in each axon (cAAD) is detailed in the caption of Figure 8. This figure shows that the MRI AAD values overestimate the AAD values extracted from histology even after correcting the histological values for the number of spins in each cylindrical axon. Clearly, this overestimation appears to be the largest in the case of the *les* SCs and minimal for the two control groups. This figure shows that the AADs are clustered and that QSI show very small ADD changes for the *taiep* group. In the *les* group, however, the QSI data show significant increases in the AADs of all ROIs compared to controls, which is not observed in the average axon size extracted from EM.

Table A4 presents the effect of diffusion time of the apparent RaDis extracted from the different QSI experiments performed on five different SCs. Both *taiep* and *les* mutants and their littermates were studied along with a control mature SC of a three-month-old rat. The results clearly show that diffusion time had a marginal effect of the apparent RaDis extracted for the mature control SC. The maximal effect, namely the larger increase in the apparent RaDis extracted from the QSI with the increase in diffusion, is observed for the SC of the 28-day-old *les* mutant that has the maximal myelin loss.

## 4. Discussion

WM-associated disorders are devastating diseases and finding MRI biomarkers that can detect this pathology at the early stage of the disease may benefit patients’ management. In the present study, we used QSI and DTI, collected with the same sequence, resolution and timing parameters, to study the SCs in the early phase of two inherited WM disorders that differ in the severity of their WM pathology [4,5,6,7,8,9,10]. In both pathologies, we observed an increase in radial displacement concomitantly with a decrease in PZD and in the FA, indicative of myelin abnormality. The changes in the diffusion MRI indices follow the known severity of the pathologies. The results show that both QSI and DTI can detect the severe *les* pathology, but QSI shows much higher statistical significance between the *les* and their littermates. However, only the QSI indices can detect the milder and clinically more relevant *taiep* pathology at this early stage. Note that at 28 days, normal maturation is still not complete in the rat spinal cord [25] so a mild pathology, as in the *taiep* SCs that had primarily mild hypo-myelination, should be difficult to detect and in fact, the FA, computed from the DTI experiments, failed to do so. Since the DTI and QSI data were collected with the same pulse sequence and with similar timing parameters—and acquisition was performed to ensure similar signal-to-nose ratios (SNRs), at least for the b_0_ images of each method—we can ascribe the higher sensitivity of QSI compared to DTI to the higher diffusion weighting of the former. Diffusion MR experiments are filter experiments, and having a higher diffusion weighting means that the MR signal will be more sensitive to slow-diffusing water populations. Consequently, one may anticipate such diffusion MR data to show higher sensitivity to restricted water populations. Using the experimental parameters utilized in the present study, the QSI images are sensitive to slow-diffusing populations that have relatively long T2, such as intra-axonal water, since a TE of 65 ms was used. With such TE, the contribution of myelin water, whose TE is smaller than 20 ms [62], is marginal. Our QSI and DTI results clearly demonstrate the benefit of using high diffusion weighting in diffusion MRI when the aim is early detection of mild myelin-associated disorders. So, even though water diffusion in WM is only an indirect measure of myelin content [62,63], the use of RaDis as obtained by QSI increases our ability to detect the very mild and clinically relevant pathology in the SCs of the *taiep* rats at early stages of the disease. Note that a recent meta-analysis of different MRI measures of myelin has listed radial diffusivity among the several MR indices that are sensitive to myelin content, but failed to identify a single marker that is more sensitive than other indices. Note, however, that T2 was not tested in this analysis [64].

The QSI data show that, in both pathologies, there is an increase in the RaDis, that at long TE, is believed to represent mostly intra-axonal water and hence the average axon diameter (AAD). Indeed, the AADs extracted from the QSI data in the present study are about 2.1–2.4 microns for the SCs of the control groups. These values are somewhat higher than the values extracted from the EM images of the control groups, from which an average axon diameter range of 1.0–1.5 microns was extracted. The differences between the QSI and EM results are even smaller if we correct the EM values to the number of spins, which is in fact what the MRI measures. After this correction, the cAAD values extracted from histology for the control groups are in the range of 1.0–1.9 microns. Note that our histological findings are similar to the findings in recent morphometry studies performed on rat and even human SCs [65,66]. The fact that the AADs extracted from the diffusion MRI data are indeed larger from the histological values in the control SCs may be ascribed to two major effects. The two main effects are exchange and the structural resolution limit of the diffusion MRI experiments. The structural resolution limit is more important when smaller compartments are being characterized by diffusion MR experiments. It originates from the insufficient strength of the diffusion gradients used as derived by Nilsson et al. [67]. This is one of the main reasons for the fact that in most clinical MRI studies devoted to measuring axonal diameter, the values extracted from the diffusion MR data are found to be significantly larger than the values derived from histology [65,66,67,68,69,70,71,72,73]. The AAD values, extracted from many of the diffusion MRI studies devoted to measure this parameter in the SC, are even larger than the differences found in the present study that used relatively strong pulse gradients. The QSI study that, indeed, was able to reproduce the best histological axon diameters was published by Ong et al. [74]. This is not surprising since in these q-space diffusion MRS experiments, axon diameters were extracted with extremely high diffusion weighting and short gradient pulses. Gradient pulses of up to 48,000 mT/m were used which, even with a relatively short gradient pulse length of 400µs, resulted in a *q_max_* of 8173 cm^−1^. In the present study, in the SCs of 28-day-old control groups we observe, even though maturation is not complete, relatively robust myelin sheaths. This implies that in these groups of SCs, exchange should play a less dramatic role and indeed the differences between the AAD values extracted from histology and MRI are modest.

Inspection of the EM images of the *les* SCs show nearly complete loss of the myelin sheaths. In the *taiep* SCs a less severe but still significant reduction in the myelin thickness is observed with an increase in the g-ratio. These changes in myelin thickness are, however, not accompanied by a significant increase in the axon diameter, as suggested from the QSI data. Indeed, even in the *les* SCs where we observe about a 50% increase in the RaDis and hence in the AAD, the quantitative analysis of the EM data shows no significant increase in axonal diameter. For the *taiep* SCs, where a much smaller increase is found in the RaDis, we observe small increase and decrease in the mean axon diameter as computed from EM for the three ROIs examined. So, clearly the increase in the RaDis computed from QSI under the experimental parameters used (i.e., diffusion time of 50 ms, TE of 65 ms and high diffusion weighting) best reflects the myelin thickness in the diseased WM and not the AAD. Thinner myelin sheaths correlate with increased RaDis and hence the MRI-computed average axon diameters. The reason for this observation may well be the exchange of the restricted intra-axonal water into the extra-axonal space during the diffusion time. Such exchange should be faster for axons that have thinner myelin sheaths. This implies that when computing the average axon diameter from diffusion MRI data not collected with extremely short diffusion time, i.e., less than 10 ms, is it more accurate to refer to the apparent AAD. This is especially true when following disorders with a significant decrease in myelin thickness or myelin losses where exchange may play a much more dominant role.

To verify the effect of exchange even further, we collected QSI data with different diffusion times (i.e., 30, 50 and 75 ms at TE of 90 ms) on five SCs representing the four groups studied in the present study and one SC of a 3-month-old control rat representing a normal, fully mature SC. The AADs extracted from all these QSI experiments are presented in Table A4. These data show that clearly, there is practically no effect on the apparent AAD extracted from QSI in the case of the fully mature SC when the diffusion is increased from 30 to 75 ms. Even in the non-mature 28-day-old control SCs, we see only marginal changes of about 5% increase in the extracted AAD upon the 2.5-fold increase in the diffusion time. As expected, in the *taiep* SC, we observe some effect, and at longer diffusion times, somewhat larger apparent AADs are extracted. Clearly, the maximal effect of the diffusion time on the apparent AADs was observed in the case of the SCs of the 28-day-old *les* rats that have no myelin. Up to a 35% increase was observed in the extracted axon diameters. These results corroborate our assumption that, indeed, exchange becomes more important in the case of severe myelin loss and an increase in the g-ratio in the damaged WM emphasizes the need to use the term apparent AAD (aAAD) when using QSI to measure axon diameter in highly hypomyelinated WM.

The accuracy of axon diameter estimates by diffusion MRI methods is still under some debate, especially when clinical MRI scanners are used [73,75,76,77,78]. One of the main reasons for the above observation is the insufficient gradient strength of clinical MRI scanners. It became clear, however, that increasing the gradient strength of clinical MRI scanners alleviated this issue at least partially [67,78]. QSI was previously used only by a few groups in clinical settings [19,20,27,28,79,80,81,82], and increasing the gradients on clinical MRI scanners to 300 mT/m [83], or even to 500 mT/m [84], may even further facilitate the applications of QSI technique in clinical studies.

Ex vivo diffusion MRI has advantages and allows obtaining such images with relatively high resolution, high SNR and relatively less motion artifacts. In addition, ex vivo diffusion MRI allows for direct comparison with histology; however, it does have limitations. The main limitation is that the extrapolation of results obtained in ex vivo formalin-fixed SCs to in vivo situations should be performed cautiously. However, radial diffusivity and FA are clearly among the most robust MR parameters that one can measure in excised tissues. Additional limitations of the present study are the relatively small groups studied and the use of relatively simple, non-parametric diffusion MRI methodologies. Indeed, in recent years, more sophisticated diffusion methods and modeling have been presented [38,39,40,41], some of which were used to study the microstructure and axon size in the SC [85,86,87,88]. Another limitation is that QSI requires strong gradients, which make it less suitable for clinical application, however recent hardware developments [83,84] may, partially, alleviate this problem.

## 5. Conclusions

QSI and DTI collected with similar experimental parameters were used to study, ex vivo, the SCs in the early phase of two experimental models of inherited myelin disorders that differ in their severity and results were compared with quantitative EM findings. Both QSI and DTI were able to detect the severe *les* pathology, but only QSI was able to detect the much milder but more clinically relevant pathology seen in the SCs of the *taiep* rats, demonstrating the superiority of heavy diffusion weighting for detecting mild myelin changes. The increase in the RaDis followed the severity of the pathologies. A comparison with detailed EM data, in which the axon diameter and myelin thickness were computed, shows better correspondence between the RaDis and the MRI AAD, not with the axon diameter computed from EM but rather with the myelin thickness. These results demonstrate that the axon diameter—as extracted from the RaDis computed from QSI and known to be a good approximation to the physical axon size in healthy, mature WM—overestimates the AAD obtained from histology in the case of myelin disorders. This is even more so when there is a significant decrease in myelin thickness since exchange is then more important and should be taken into consideration. The results show that the model used to describe the tissue may change in the case of severe myelin damage and the proposal is to use the term of apparent average axonal diameter (aAAD) rather than AAD in such pathologies. It appears that when there is a complete absence or severe loss of myelin, exchange should be taken into consideration if one aims at characterizing average axonal diameter without using ultrashort diffusing time diffusion MRI protocols.

## Data Availability

Data are contained within the manuscript and the Appendix A.

## Abbreviations

The following abbreviations are used in this manuscript:

AAD	Average axon diameter
AxD	Axial diffusivity
CNS	Central nervous system
DTI	Diffusion tensor imaging
EM	Electron microscopy
EPI	Echo planar imaging
FA	Fractional anisotropy
*Les*	Long–Evans Shaker
MBP	Myelin basic protein
md	Myelin-deficient
MD	Mean diffusivity
MS	Multiple sclerosis
PLP	Proteolipid protein
PZD	Probability for zero displacement
QSI	Q-space diffusion MRI
RaD	Radial diffusivity
RaDis	Radial displacement
ROI	Region of interest
SC	Spinal cord
SNR	Signal-to-noise ratio
WM	White matter

## Appendix A

**Table A1:** Values and group average values of radial displacement (RaDis in μm), the probability for zero displacement (PZD) and FA (in arbitrary units, a.u.) in the defined ROIs of the control and *taiep* spinal cords of 28-day-old rats and the calculated *p*-values.

	Displacement [µm]			Probability [a.u.]			FA [a.u]		
Type	ROI 1	ROI 2	ROI 3	ROI 1	ROI 2	ROI 3	ROI 1	ROI 2	ROI 3
**Control**	2.10 ± 0.03	2.27 ± 0.02	2.28 ± 0.06	12.22 ± 0.47	10.55 ± 0.63	11.19 ± 0.55	0.85 ± 0.04	0.80 ± 0.05	0.80 ± 0.02
*** Control**	2.10 ± 0.02	2.22 ± 0.04	2.34 ± 0.04	12.41 ± 0.73	10.98 ± 0.68	11.46 ± 0.29	0.82 ± 0.03	0.80 ± 0.04	0.80 ± 0.03
*** Control**	2.07 ± 0.03	2.31 ± 0.04	2.24 ± 0.02	12.75 ± 0.64	10.66 ± 0.16	11.32 ± 0.25	0.80 ± 0.03	0.85 ± 0.03	0.86 ± 0.02
**Control**	2.12 ± 0.05	2.23 ± 0.02	2.36 ± 0.02	12.09 ± 0.43	10.70 ± 0.23	10.28 ± 0.23	0.82 ± 0.02	0.74 ± 0.02	0.72 ± 0.06
**Control**	2.12 ± 0.04	2.30 ± 0.01	2.40 ± 0.02	12.59 ± 0.51	9.82 ± 0.34	9.88 ± 0.36	0.88 ± 0.03	0.77 ± 0.04	0.76 ± 0.02
**Average**	**2.10 ± 0.02**	**2.27 ± 0.04**	**2.33 ± 0.06**	**12.41 ± 0.27**	**10.54 ± 0.43**	**10.82 ± 0.70**	**0.83 ± 0.03**	**0.79 ± 0.04**	**0.79 ± 0.05**
*** *Taiep***	2.21 ± 0.07	2.32 ± 0.03	2.33 ± 0.03	11.53 ± 0.62	9.65 ± 0.53	10.13 ± 0.19	0.82 ± 0.07	0.75 ± 0.03	0.77 ± 0.02
*** *Taiep***	2.32 ± 0.13	2.25 ± 0.02	2.24 ± 0.03	11.07 ± 0.57	10.30 ± 0.12	10.91 ± 0.11	0.77 ± 0.05	0.77 ± 0.02	0.73 ± 0.06
***Taiep***	2.22 ± 0.09	2.37 ± 0.08	2.46 ± 0.01	11.25 ± 0.56	9.58 ± 0.24	9.27 ± 0.04	0.75 ± 0.05	0.80 ± 0.04	0.78 ± 0.03
***Taiep***	2.32 ± 0.01	2.50 ± 0.07	2.46 ± 0.01	10.47 ± 0.19	10.44 ± 0.29	9.70 ± 0.37	0.74 ± 0.06	0.73 ± 0.01	0.71 ± 0.04
***Taiep***	2.25 ± 0.06	2.36 ± 0.04	2.39 ± 0.02	10.95 ± 0.50	10.14 ± 0.13	9.65 ± 0.02	0.85 ± 0.02	0.76 ± 0.02	0.83 ± 0.02
**Average**	**2.26 ± 0.05**	**2.36 ± 0.09**	**2.38 ± 0.09**	**11.06 ± 0.39**	**10.02 ± 0.39**	**9.93 ± 0.63**	**0.79 ± 0.04**	**0.76 ± 0.02**	**0.76 ± 0.04**
***p*** **-value**	0.0015	0.0827	0.3457	0.0004	0.0811	0.0665	0.10002	0.20807	0.46531

* Spinal cords used for electron microscopy.

**Table A2:** Values and group average values of radial displacement (RaDis in μm), probability for zero displacement (PZD) and FA (in arbitrary units, a.u.) in the defined ROIs of the control and *les* spinal cords of 28-day-old rats and the calculated *p*-values.

	Displacement [µm]			Probability [a.u.]			FA [a.u]		
Type	ROI 1	ROI 2	ROI 3	ROI 1	ROI 2	ROI 3	ROI 1	ROI 2	ROI 3
**Control**	2.11 ± 0.04	2.24 ± 0.01	2.36 ± 0.02	12.33 ± 0.21	10.70 ± 0.36	9.95 ± 0.08	0.82 ± 0.02	0.76 ± 0.05	0.65 ± 0.04
*** Control**	2.25 ± 0.03	2.35 ± 0.02	2.36 ± 0.01	10.96 ± 0.37	9.63 ± 0.21	9.78 ± 0.23	0.77 ± 0.02	0.70 ± 0.03	0.72 ± 0.06
*** Control**	2.20 ± 0.04	2.42 ± 0.07	2.42 ± 0.02	11.33 ± 0.37	8.93 ± 0.26	9.58 ± 0.11	0.82 ± 0.05	0.75 ± 0.02	0.75 ± 0.03
**Control**	2.06 ± 0.06	2.44 ± 0.05	2.33 ± 0.09	12.82 ± 0.65	10.16 ± 0.52	10.71 ± 0.52	0.90 ± 0.04	0.71 ± 0.04	0.74 ± 0.05
**Control**	2.09 ± 0.03	2.32 ± 0.00	2.40 ± 0.04	12.86 ± 0.42	10.07 ± 0.17	10.54 ± 0.32	0.84 ± 0.01	0.73 ± 0.01	0.66 ± 0.02
**Average**	**2.14 ± 0.07**	**2.35 ± 0.07**	**2.37 ± 0.03**	**12.06 ± 0.78**	**9.90 ± 0.59**	**10.11 ± 0.44**	**0.83 ± 0.04**	**0.73 ± 0.02**	**0.70 ± 0.04**
***Les***	2.87 ± 0.06	3.16 ± 0.07	3.65 ± 0.09	8.30 ± 0.28	8.30 ± 0.27	6.45 ± 0.25	0.72 ± 0.03	0.60 ± 0.03	0.60 ± 0.04
***Les***	3.43 ± 0.08	3.24 ± 0.03	3.57 ± 0.09	6.82 ± 0.18	7.23 ± 0.21	6.64 ± 0.09	0.67 ± 0.04	0.62 ± 0.02	0.67 ± 0.03
*** *Les***	2.77 ± 0.08	3.09 ± 0.03	3.07 ± 0.06	8.52 ± 0.28	7.25 ± 0.26	7.49 ± 0.22	0.72 ± 0.04	0.72 ± 0.01	0.74 ± 0.04
*** *Les***	2.95 ± 0.04	3.05 ± 0.06	3.16 ± 0.05	7.96 ± 0.10	7.72 ± 0.15	7.24 ± 0.15	0.73 ± 0.03	0.69 ± 0.02	0.70 ± 0.02
***Les***	3.05 ± 0.04	3.39 ± 0.20	3.72 ± 0.08	7.63 ± 0.22	6.90 ± 0.24	6.30 ± 0.12	0.80 ± 0.01	0.67 ± 0.03	0.54 ± 0.01
**Average**	**3.01 ± 0.23**	**3.19 ± 0.12**	**3.43 ± 0.27**	**7.85 ± 0.59**	**7.48 ± 0.49**	**6.82 ± 0.46**	**0.73 ± 0.04**	**0.66 ± 0.04**	**0.65 ± 0.07**
***p*** **-values**	**0.00075**	**0.00001**	**0.00138**	**0.00006**	**0.00023**	**0.00001**	**0.00869**	**0.03070**	**0.23894**

* Spinal cords used for electron microscopy.

**Table A3:** The average axon diameters (AADs in μm), before and after correction for spin density, and myelin thickness (in μm) as extracted from EM for the three ROIs.

Sample	ROI	AAD	AAD After Correction	Myelin Thickness
**Control**	ROI 1	1.14 ± 0.06	1.39 ± 0.09	0.22 ± 0.006
	ROI 2	1.34 ± 0.10	1.76 ± 0.10	0.19 ± 0.007
	ROI 3	1.24 ± 0.12	1.63 ± 0.15	0.33 ± 0.018
**Control**	ROI 1	0.98 ± 0.05	1.02 ± 0.06	0.21 ± 0.010
	ROI 2	1.40 ± 0.11	1.94 ± 0.14	0.18 ± 0.010
	ROI 3	1.06 ± 0.11	1.43 ± 0.15	0.28 ± 0.020
***Taiep***	ROI 1	1.38 ± 0.07	1.84 ± 0.09	0.12 ± 0.003
	ROI 2	1.11 ± 0.09	1.64 ± 0.16	0.12 ± 0.003
	ROI 3	1.34 ± 0.11	1.88 ± 0.15	0.16 ± 0.010
***Taiep***	ROI 1	1.13 ± 0.12	1.14 ± 0.07	0.11 ± 0.003
	ROI 2	0.83 ± 0.06	0.80 ± 0.08	0.11 ± 0.004
	ROI 3	1.46 ± 0.14	2.20 ± 0.17	0.15 ± 0.007
**Control**	ROI 1	1.63 ± 0.09	2.70 ± 0.15	0.24 ± 0.010
	ROI 2	1.23 ± 0.10	1.81 ± 0.14	0.30 ± 0.013
	ROI 3	1.71 ± 0.14	3.08 ± 0.22	0.35 ± 0.018
**Control**	ROI 1	1.33 ± 0.07	1.74 ± 0.11	0.23 ± 0.007
	ROI 2	1.36 ± 0.10	1.84 ± 0.13	0.29 ± 0.012
	ROI 3	1.72 ± 0.13	2.95 ± 0.21	0.33 ± 0.017
***Les***	ROI 1	1.05 ± 0.05	0.92 ± 0.05	0.00 ± 0.000
	ROI 2	1.58 ± 0.10	2.10 ± 0.16	0.00 ± 0.000
	ROI 3	1.79 ± 0.12	2.85 ± 0.19	0.00 ± 0.000
***Les***	ROI 1	0.94 ± 0.05	0.87 ± 0.06	0.00 ± 0.000
	ROI 2	1.31 ± 0.10	1.57 ± 0.13	0.00 ± 0.000
	ROI 3	1.99 ± 0.14	3.48 ± 0.22	0.00 ± 0.000

**Table A4:** The effect of the diffusion time (∆) at constant TE, on the apparent radial displacement (RaDis in μm) as extracted from the QSI for the three ROIs.

ROI	Protocol TE/δ/Δ (ms)	WT/*Les*-28-Day-Old	*Les*-28-Day-Old	WT/*Taiep*-28-Day-Old	*Taiep*-28-Day-Old	WT/*Taiep*-3-Month-Old
**ROI 1**	90/2/30	2.11 ± 0.02	2.54 ± 0.05	2.15 ± 0.04	2.17 ± 0.03	2.08 ± 0.02
	90/2/50	2.20 ± 0.04	3.04 ± 0.08	2.31 ± 0.05	2.19 ± 0.04	2.05 ± 0.03
	90/2/75	2.31 ± 0.05	3.50 ± 0.22	2.26 ± 0.06	2.39 ± 0.06	2.07 ± 0.02
**ROI 2**	90/2/30	2.16 ± 0.01	2.64 ± 0.11	2.23 ± 0.03	2.18 ± 0.01	2.12 ± 0.01
	90/2/50	2.29 ± 0.02	3.21 ± 0.14	2.26 ± 0.06	2.16 ± 0.01	2.12 ± 0.02
	90/2/75	2.39 ± 0.06	3.58 ± 0.12	2.22 ± 0.01	2.42 ± 0.07	2.15 ± 0.03
**ROI 3**	90/2/30	2.23 ± 0.03	2.76 ± 0.05	2.26 ± 0.03	2.20 ± 0.01	2.16 ± 0.01
	90/2/50	2.31 ± 0.03	3.46 ± 0.08	2.31 ± 0.06	2.20 ± 0.03	2.16 ± 0.01
	90/2/75	2.40 ± 0.04	4.12 ± 0.09	2.58 ± 0.02	2.38 ± 0.04	2.24 ± 0.03
